# Stress and anxiety among Medical residents working during the covid-19 pandemic

**DOI:** 10.1192/j.eurpsy.2023.1680

**Published:** 2023-07-19

**Authors:** I. Hanine, M. Chtibi, S. Belbachir, A. Ouanass

**Affiliations:** 1 Military hospital Mohammed V of Rabat, Rabat; 2 Psychiatry, Arrazi University psychiatric hospital of Sale, Morocco, Sale, Morocco

## Abstract

**Introduction:**

Covid-19 is believed to be one of the most impactful events of the 21’s century,

Pressure related to this pandemic was put on every of the health system especially residents.

Medical residents whose hierarchical position is particular, in the framework of their training they are subjected to an increased level of stress due to the constant pressure of training and the current challenges of being in the front line of the pandemic.

**Objectives:**

The aim of our study is to evaluate the presence of stress in medical residents.

**Methods:**

Using a self-evaluation questionnaire with two parts, the first exploring age, sexe, history of medical, surgical and psychiatric disorders the second part exploring stress with the French version of PSS-10 (preveived stress scale).

**Results:**

Concerning our descriptive statistcs: among our 140 residents, percentage of male and female residents were almost equal with 2,85% of them already had a record of an anxiety disorder’s follow-up, 71,4% had a moderate stress level and 8,6% had high stress level.

**Conclusions:**

Our study led us to the following conclusion, stress is a component that affects the quality and the work performed by the vast majority of health care workers.

**Disclosure of Interest:**

None Declared

